# Tlr2/4‐Mediated Hyperinflammation Promotes Cherubism‐Like Jawbone Expansion in Sh3bp2 (P416R) Knockin Mice

**DOI:** 10.1002/jbm4.10562

**Published:** 2021-10-30

**Authors:** Yasuyuki Fujii, Nelson Monteiro, Shyam Kishor Sah, Homan Javaheri, Yasuyoshi Ueki, Zhichao Fan, Ernst J Reichenberger, I‐Ping Chen

**Affiliations:** ^1^ Department of Oral Health and Diagnostic Sciences, School of Dental Medicine University of Connecticut Health Farmington CT USA; ^2^ Department of Biomedical Sciences and Comprehensive Care Indiana University School of Dentistry Indianapolis IN USA; ^3^ Indiana Center for Musculoskeletal Health Indiana University, School of Medicine Indianapolis IN USA; ^4^ Department of Immunology, School of Medicine University of Connecticut Health Farmington CT USA; ^5^ Center for Regenerative Medicine and Skeletal Development, Department of Reconstructive Sciences University of Connecticut Health Farmington CT USA

**Keywords:** GENETIC ANIMAL MODELS, CHERUBISM, OSTEOIMMUNOLOGY, JAW ABNORMALITIES, NEUTROPHIL EXTRACELLULAR TRAPS

## Abstract

Cherubism (CBM), characterized by expansile jawbones with multilocular fibrocystic lesions, is caused by gain‐of‐function mutations in SH3 domain‐binding protein 2 (*SH3BP2*; mouse orthologue *Sh3bp2*). Loss of jawbone and dental integrity significantly decrease the quality of life for affected children. Treatment for CBM is limited to multiple surgeries to correct facial deformities. Despite significant advances made with CBM knockin (KI) mouse models (*Sh3bp2* ^*KI/KI*^), the activation mechanisms of CBM lesions remain unknown because mutant mice do not spontaneously develop expansile jawbones. We hypothesize that bony inflammation of an unknown cause triggers jawbone expansion in CBM. To introduce jawbone inflammation in a spatiotemporally controlled manner, we exposed pulp of the first right mandibular molar of 6‐week‐old *Sh3bp2* ^*+/+*^, *Sh3bp2* ^*KI/+*^, and *Sh3bp2* ^*KI/KI*^ mice. Bacterial invasion from the exposed pulp into root canals led to apical periodontitis in wild‐type and mutant mice. The pathogen‐associated molecular patterns (PAMPs)‐induced inflammation of alveolar bone resulted in jawbone expansion in *Sh3bp2* ^*KI/+*^ and *Sh3bp2* ^*KI/KI*^ mice. CBM‐like lesions developed exacerbated inflammation with increased neutrophil, macrophage, and osteoclast numbers. These lesions displayed excessive neutrophil extracellular traps (NETs) compared to *Sh3bp2* ^*+/+*^ mice. Expression levels of *IL‐1β*, *IL‐6*, and *TNF‐α* were increased in periapical lesions of *Sh3bp2* ^*+/+*^, *Sh3bp2* ^*KI/+*^, and *Sh3bp2* ^*KI/KI*^ mice and also in plasma and the left untreated mandibles (with no pulp exposure) of *Sh3bp2* ^*KI/KI*^ mice, suggesting a systemic upregulation. Ablation of Tlr2/4 signaling or depletion of neutrophils by Ly6G antibodies ameliorated jawbone expansion induced by PAMPs in *Sh3bp2* ^*KI/KI*^ mice. In summary, successful induction of CBM‐like lesions in jaws of CBM mice is important for studying initiating mechanisms of CBM and for testing potential therapies. Our findings further emphasize a critical role of host immunity in the development of apical periodontitis and the importance of maintaining oral health in CBM patients. © 2021 The Authors. *JBMR Plus* published by Wiley Periodicals LLC on behalf of American Society for Bone and Mineral Research.

## Introduction

Cherubism (CBM, OMIM#118400) is a rare genetic inflammatory bone disorder characterized by expansile jawbones with multilocular fibrocystic lesions.^(^ ^1^ ^)^ The age of onset is typically at 2 to 5 years of age. Submaxillary and submandibular lymph node swelling, a sign of ongoing inflammation, is frequently noted in CBM patients.^(^ ^2^, ^3^ ^)^ Facial deformity and dental abnormalities cause functional and psychological distress in affected children. Treatment is limited to conservative oral care, local curettage of lesions, and multiple surgeries to correct facial deformities in severe cases.^(^ ^3^, ^4^ ^)^ In milder cases, CBM lesions often regress spontaneously after puberty. Understanding the pathogenesis of CBM is a prerequisite for better clinical care and intervention.

Missense mutations in *SH3BP2* have been identified as cause for the autosomal dominant form of CBM.^(^ ^5^ ^)^ CBM mutations protect SH3BP2 protein from tankyrase‐mediated degradation leading to SH3BP2 accumulation and increased proto‐oncogene tyrosine‐protein kinase, spleen tyrosine kinase, PLCγ1/γ2 (Phospholipase C gamma 1/2), and vav Guanine nucleotide exchange factor signaling.^(^ ^6^, ^7^, ^8^, ^9^ ^)^ Studies of knockin (KI) mice expressing mutant SH3BP2 protein (P416R or G418R) showed that *Sh3bp2* ^*KI/KI*^ mice develop osteopenia, and autoinflammatory lesions in long bones, craniofacial bones, and soft tissues. However, existing mouse models for CBM do not spontaneously develop jawbone expansions, a major phenotype of human CBM. This species difference prevented experimental studies on lesion development for CBM and drug testing for treatment or prevention.

It has been hypothesized that PAMPs and damage‐associated molecular patterns (DAMPs) in the oral cavity may contribute to jaw‐specific autoinflammation in CBM.^(^ ^10^ ^)^ Bacteria, endotoxins, and metabolic byproducts from caries egressing into root canals and into periapical tissues stimulate the host immune system to produce an inflammatory response that results in apical periodontitis.^(^ ^11^ ^)^ Histologically, periapical lesions may show bacteria colonies surrounded by dense concentration of polymorphonuclear leukocytes (PMNs) and inflammatory lesions filled with plasma cells, lymphocytes, neutrophils, monocytes, macrophages, and mast cells.^(^ ^12^ ^)^ Cytokines released by host cells, such as interleukin 1 (IL‐1), IL‐6, and tumor necrosis factor α (TNF‐α), etc., mediate multiple immunologic and non‐immunologic events, which contribute to periapical inflammation in apical periodontitis.^(^ ^13^ ^)^ *Sh3bp2* ^KI/KI^ mice exhibit increased TNF‐α serum levels, and macrophage and osteoclast numbers in bone lesions.^(^ ^6^, ^14^ ^)^ Whether CBM mutations in SH3BP2 exacerbate apical periodontitis induced by dental infection and whether PAMPs are involved in the initiation of CBM lesions has not been studied due to a lack of suitable animal models.

Although clinical studies have identified macrophages and osteoclasts in human CBM lesions,^(^ ^15^, ^16^ ^)^ the role of neutrophils in CBM has never been studied although SH3BP2 is required for neutrophil activation.^(^ ^17^ ^)^ Neutrophils are the most abundant type of white blood cells and predominant at the initial inflammatory response. Upon activation, neutrophils kill pathogens by phagocytosis, degranulation, and formation of neutrophil extracellular traps (NETs), which are web‐like structures decorated with decondensated DNA and bactericidal proteins.^(^ ^18^ ^)^ Although NETs kill microorganisms, excessive NETs can cause tissue damage by aberrantly stimulating immune cells to secrete inflammatory cytokines.^(^ ^18^, ^19^ ^)^

To introduce jawbone inflammation reliably and consistently, we induced apical periodontitis in mice by pulp exposure of one lower molar. For the first time we could show that heterozygous and homozygous *Sh3bp2* KI mice with a P416R mutation develop CBM‐like expansile jawbones in a predictable location and time course. This model further elucidates the contribution of neutrophil‐derived events in CBM‐like lesions.

## Materials and Methods

### Mice

*Sh3bp2* (P416R) KI mice have been reported.^(^ ^6^ ^)^ *Tlr2* ^*−/−*^ mice and *Tlr4* ^*lps–del/lps–del*^ mice were obtained from the Jackson Laboratory (Bar Harbor, ME, USA) and were crossed to obtain Tlr2 and Tlr4 homozygous double knockout (KO) mice (*Tlr2/4* ^*KO/KO*^ mice). *Tlr2/4* ^*KO/KO*^ mice were crossed with *Sh3bp2* ^*KI/+*^ mice. Triple mutant mice (*Sh3bp2* ^*+/+*^ *Tlr2/4* ^*KO/KO*^ and *Sh3bp2* ^*KI/KI*^ *Tlr2/4* ^*KO/KO*^) were generated by crossing *Sh3bp2* ^*KI/+*^ *Tlr2/4* ^*KO/KO*^ mice and *Sh3bp2* ^*KI/+*^ *Tlr2/4* ^*KO/KO*^ mice. All strains were on C57BL/6J background housed in a specific pathogen‐free environment with a normally complex microflora. We have conformed to the Animals in Research: Reporting In Vivo Experiments (ARRIVE) guidelines.

### Pulp exposure in mice

To induce jawbone infection in a defined location and time course, the dental pulp of right mandibular first (1st) molars of 6‐week‐old male and female mice was exposed with a #1/4 round carbide bur powered by an electric handpiece (AEU‐20; Tulsa Dental, Tulsa, OK, USA) under a microscope (Carl Zeiss Microscopy, Inc., Dublin, CA, USA) after ketamine (100 mg/kg) and xylazine (10 mg/mL) anesthesia via intraperitoneal injection. At indicated time points, peripheral blood was collected for ELISA assays and mandibles were harvested from naïve (no pulp exposed) mice and mice with pulp exposure for image analysis, histology, or RNA isolation.

### Faxitron and micro–computed tomography imaging analysis

**Fig. 1:**
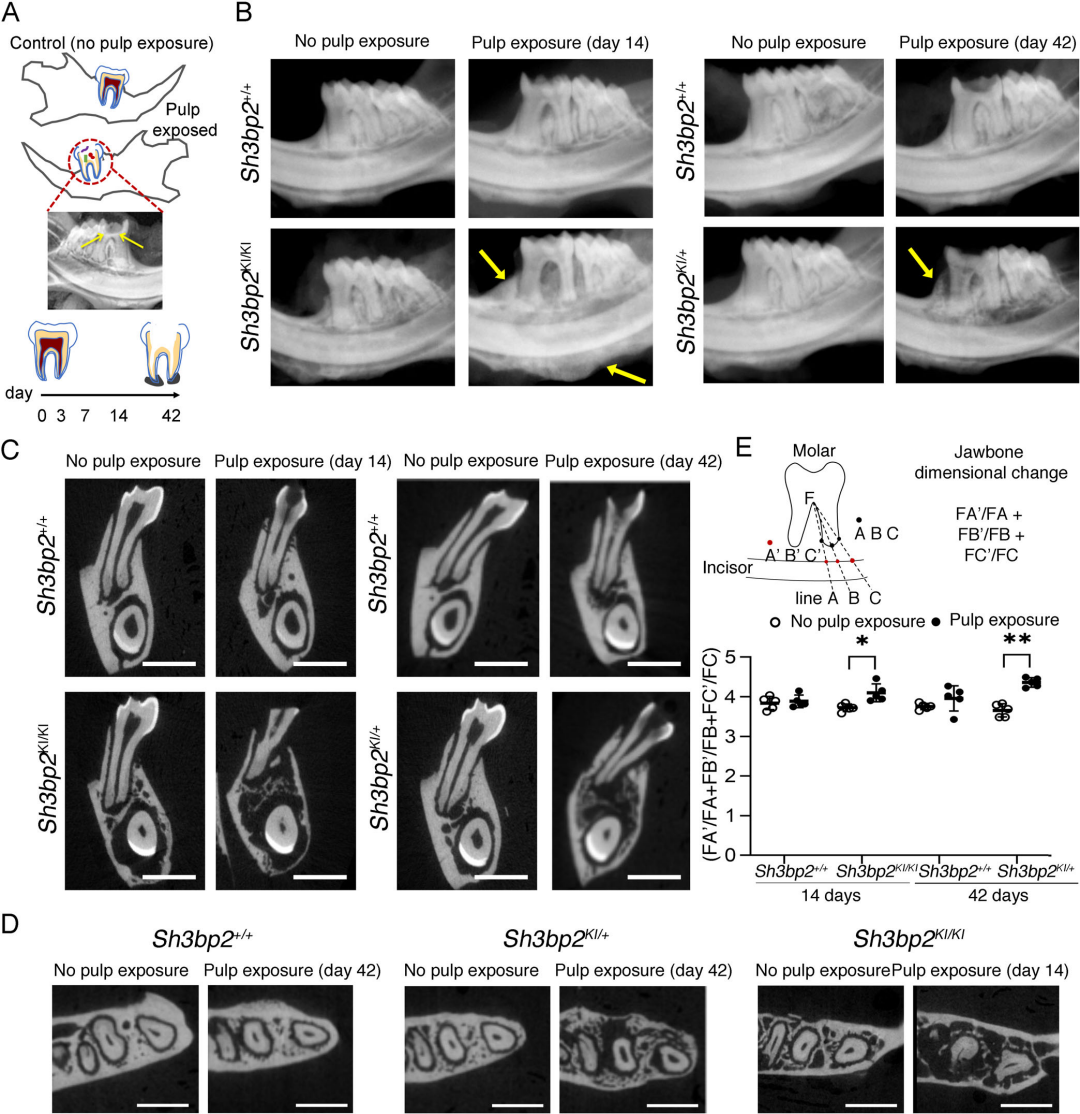
CBM‐like jawbone expansion in *Sh3bp2* mutant mice. (*A*) Timeline of mandible collection with or without pulp exposure of right 1st molar. Faxitron image confirmed the pulp access (yellow arrows). (*B*) Representative Faxitron images of expansile jawbones in *Sh3bp2* ^*KI/+*^ and *Sh3bp2* ^*KI/KI*^ female mice. Yellow arrows indicate jawbone expansion. (*C*) Coronal planes and (*D*) axial planes of μCT images. Scale bar = 1 mm. (*E*) Quantification of jawbone dimensions by measuring distances from furcation of 1st molars to the top margin of incisors (FA′, FB′, FC′) and normalizing to distances from furcation to the end of root tips (FA, FB, FC) along lines A, B, and C. Data are presented as mean ± SD. Each dot in graphs represents a single biological sample (*n* = 5 per group). *p < 0.05, **p < 0.01 indicate statistical significance by two‐way ANOVA followed by Sidak correction. F = furcation.

Radiographs of mandibles were obtained by a MX20 Radiography System (Faxitron X‐Ray, Tucson, AZ, USA) and were used to evaluate jawbone expansion after pulp exposure. Reference lines and landmarks were defined as shown in Fig. 1*E*. Comparing the mandibular total volume of naïve and pulp‐exposed mice did not reflect the dimensional changes of mandibles because of large variability. We therefore decided to measure parameters that changed in expansile mandibles (FA′, FB′, FC′) normalized to the measurements that would not be changed due to pulp exposure, which are the length of roots (FA, FB, FC). Radiographic analysis was performed with Photoshop CC (Adobe, San Jose, CA, USA). Micro–computed tomography (μCT) scanning was performed in a mCT20 (SCANCO Medical AG, Brüttisellen, Switzerland).

### Histological analysis

Mouse mandibles were fixed in 4% paraformaldehyde followed by decalcification with 14% ethylenediamine tetraacetic acid (EDTA) solution under gentle shaking in a cold room. Decalcified samples were embedded in paraffin and sectioned to a thickness of 5 μm (Leica microtome; Leica Biosystems Inc., Buffalo Grove, IL, USA). Sections were deparaffinized and subjected to hematoxylin and eosin (H&E) staining and immunohistochemistry with antibodies of LPS core (1:100, HM6011; Hycult Biotech, Uden, Netherlands), NIMPR14 (1:100; sc‐59338; Santa Cruz Biotechnology, Santa Cruz, CA, USA), CD68 (1:500; aa312‐326; Lifespan Biosciences [LSBio], Seattle, WA, USA), cathepsin K (1:50; sc‐48353; Santa Cruz Biotechnology), citrullinated histone 3 (citH3) (1:50; ab5103; Abcam, Cambridge, MA, USA), myeloperoxidase (MPO) (1:40; af3667; R&D Systems, Minneapolis, MN, USA). Sections were imaged in an automated slide scanner (Axio Scan Z.1; Carl Zeiss Microscopy, Inc.). Quantification of macrophages and osteoclasts were performed by Adobe Photoshop and Digimizer image analysis software (MedCalc Software Ltd, Ostend, Belgium). The number of macrophages were counted and normalized to periapical surface area that included furcation, the area between two roots with bottom margin ending at the superior border of incisor. The surface area of osteoclasts was normalized to the perimeter of alveolar bone surrounding the mandibular first molars. The signals of MPO, citH3, and colocalization of MPO and citH3 were normalized to 4,6‐diamidino‐2‐phenylindole (DAPI) signal by the Fiji Image J software package (NIH, Bethesda, MD, USA; https://imagej.nih.gov/ij/). Three fields (450 μm × 335 μm) covering furcation and periapical area of mesial and distal roots from each mouse were measured.

### RNA analysis

Mandibles were dissected out and the mesial and distal sides of the first molar and the incisor were removed and transferred to RNAlater (Qiagen, Valencia, CA, USA). The first molar with surrounding bone was immediately frozen in liquid nitrogen. The frozen bone was crushed using a cryogenic tissue pulverizer (Thermo Fisher Scientific, Waltham, MA, USA). Bone powder was immersed in TRIzol (Invitrogen, Carlsbad, CA, USA) and homogenized (Ultra‐Turrax, IKA Werke GmbH & Co. KG, Staufen im Breisgau, Germany). Total RNA was isolated using Direct‐zol RNA kit (Zymo Research, Irvine, CA, USA). DNAse I–treated RNA was subjected to Superscript II reverse transcriptase (Invitrogen) according to the manufacturer's instructions. qPCR using SsoAdvanced Universal SYBR Green Supermix (Bio‐Rad Laboratories, Hercules, CA, USA) was performed in a CFX connect Real‐Time System (BioRad Laboratories). PCR efficiency was optimized and primer specificity was confirmed by melting curve analysis. Expression levels of genes were calculated using the 2^−∆∆Ct^ method and normalized to *18S* as endogenous control. The primer sequences used are shown in Supplemental Table S1.

### Plasma ELISA assays

Peripheral blood from submandibular veins of mice was collected in EDTA‐treated tubes (Becton, Dickinson and Company, Franklin Lakes, NJ, USA) using animal lancets (GoldenRod; Braintree Scientific, Inc., Braintree, MA, USA). Plasma was immediately prepared by centrifugation at 1000*g* for 15 minutes and stored at −80°C until use. ELISA assays using a mouse IL‐1β kit (R&D Systems), mouse IL‐6 kit (R&D Systems), and MPO kit (Thermo Fisher Scientific) were performed following manufacturers' instructions.

### Flow cytometry of blood cells and cell sorting of bone marrow cells

Peripheral blood from the submandibular vein (100 μL/mouse) was collected into heparin‐coated (cat# 63323‐540‐31; Fresenius Kabi USA LLC, Grand Island, NY, USA) tubes on ice using animal lancet (GoldenRod; Braintree Scientific, Inc.). To avoid endocytosis of monocyte marker CD115 we put samples on ice during all procedures. Red blood cells (RBCs) were lysed by adding 900 μL ice‐cold deionized water for 30 seconds. RBC lysis was stopped by adding 100 μL ice‐cold 10× PBS. Additional lysis may be needed depending on whether RBCs were lysed entirely or not. Samples were washed with ice‐cold PBS (5 minutes, 4°C) and resuspended in 100 μL ice‐cold PBS without Ca^2+^ and Mg^2+^ (cat# 10010049; Gibco, Grand Island, NY, USA) before antibody staining. Mouse bone marrow (BM) cells were flushed out from femurs and tibias using ice‐cold PBS (Gibco) and filtered through 70‐μm cell strainers (Thermo Fisher Scientific). BM cells were counted and responded at a concentration of 1 × 10^6^ cells/mL.

RBC‐lysed blood and BM cells (200 μL) were stained with L/D Zombie yellow (1:1000; BioLegend, San Diego, CA, USA) on ice for 15 minutes for analysis of viability. Fcγ receptors were blocked by mouse FcR Blocking Reagent (cat# 130‐092‐575; Miltenyi Biotec, Bergisch Gladbach, Germany) for 15 minutes and surface antigens on cells were stained for 30 minutes on ice with 1.25 μg/mL Alexa Fluor 700 (cat# 103128; BioLegend) conjugated anti‐mouse CD45 antibody, 2 μg/mL Brilliant Violet 650 conjugated anti‐mouse Ly6G antibody (cat# 127641; BioLegend), and 2 μg/mL allophycocyanin conjugated anti‐mouse CD115 antibody (cat# 135510; BioLegend). Forward‐and side‐scatter parameters were used for the exclusion of doublets from the analysis. Cell fluorescence was assessed with an LSRII (BD Biosciences, San Jose, CA, USA) and was analyzed with FlowJo, version 10.6 (FlowJo, LLC, Ashland, OR). L/D Zombie yellow‐low and CD45‐positive cells were identified as live leukocytes. Ly6G‐positive and CD115‐negative live leukocytes were identified as neutrophils (Fig. 4*B*). Ly6G‐negative and CD115‐positive live leukocytes were identified as monocytes (Fig. 4*B*). Ly6G‐positive cells were collected by a cell sorter (BD FACSAria II; BD Biosciences) and subjected to immunoblots.

### Immunoblot

We prepared whole‐cell lysates from the sorted Ly6G‐positive bone marrow neutrophils using radioimmunoprecipitation assay (RIPA) buffer (150mM NaCl, 50mM Tris, 1% NP40, 0.5% deoxycholate, and 0.1% SDS) containing Halt protein protease and phosphatase inhibitor cocktail (Thermo Fisher Scientific). The concentration of protein was determined by bicinchoninic acid (BCA) protein assay (Thermo Fisher Scientific). For immunoblotting assays the protein lysates were normalized to total protein concentration and the samples were loaded onto SDS‐PAGE gels in 6× loading buffer. Samples were transferred from SDS‐PAGE gels onto polyvinylidene fluoride (PVDF) membranes (BioRad Laboratories) using a semi‐dry transfer apparatus (BioRad Laboratories). The membranes were incubated overnight with anti‐SH3BP2 antibody (1:1000; SH3BP2 isoforms detected between 65 and 80 kDa; cat # sc‐8896; Santa Cruz Biotechnology). Primary antibody was diluted in Tris‐buffered saline containing 0.1% Tween‐20 and 5% skim milk. After incubation with primary antibodies, the membranes were washed in Tris‐buffered saline/Tween buffer and then incubated with peroxidase‐conjugated anti‐goat IgG secondary antibody (1:5000; sc‐2020; Santa Cruz Biotechnology) in Tris‐buffered saline/Tween buffer containing 5% skim milk for 1 hour. Protein bands were detected by enhanced chemiluminescent detection reagent (Azure Biosystems, Dublin, CA, USA) and visualized by an Azure c600 imaging system (Azure Biosystems).

### Study approval

Animal experiments were approved by the University of Connecticut Health IACUC (protocol #101961).

### Statistics

Statistical analysis was performed by Student's *t* test (between two groups) and one‐way or two‐way ANOVA followed by Sidak correction (for multiple comparisons) using Prism 8 software (GraphPad Software, Inc., La Jolla, CA, USA); *p* < 0.05 was considered statistically significant. We did not exclude any data points.

## Results

### CBM‐like jawbone phenotype in heterozygous and homozygous *Sh3bp2* mutant mice

PAMPs in the root canal system can cause apical periodontitis (inflammation in bone and tissues surrounding the root tips).^(^ ^11^ ^)^ To examine the consequences of bony inflammation in mice with or without the CBM mutation in SH3BP2, we exposed dental pulp of right mandibular first molars in 6‐week‐old male and female *Sh3bp2* ^*+/+*^, *Sh3bp2* ^*KI/+*^, and *Sh3bp2* ^*KI/KI*^ mice and examined right (pulp exposure) and left (no pulp exposure) mandibles at 3, 7, 14, and 42 days after dental drilling (Fig. 1*A*). The presence of bacteria/endotoxin in root canals and periapices was confirmed by anti‐LPS core immunohistochemistry (Supplemental Fig. S1 *A*). Male and female mice were equally affected by pulp exposure. After an initial loss of body weight (before day 3), *Sh3bp2* ^*+/+*^ and *Sh3bp2* ^*KI/+*^ mice continued to gain body weight, suggesting these mice tolerated the dental procedure well. *Sh3bp2* ^*KI/KI*^ mice kept losing weight for 21 days after pulp exposure (Supplemental Fig. S1 *B*).

Faxitron imaging showed that all mice developed apical periodontitis (radiolucent periapical lesions associated with root tips of 1st molars) 3 to 14 or 3 to 42 days after pulp exposure (Fig. 1*B*, Supplemental Fig. S1 *C*). More severe inflammatory bone loss was observed in *Sh3bp2* ^*KI/KI*^ and *Sh3bp2* ^*KI/+*^ mice in comparison to their *Sh3bp2* ^*+/+*^ littermates (Fig. 1*C*,*D*). In addition, significant jawbone expansion vertically and horizontally was observed in *Sh3bp2* ^*KI/KI*^ and *Sh3bp2* ^*KI/+*^ mice 14 and 42 days after pulp exposure, respectively, by Faxitron and μCT image analysis (Fig. 1*B*–*E*). In *Sh3bp2* mutant mice the expansile lesions caused a significant increase in the distances between root tips of molars and underlying incisors (Fig. 1*B*,*C*). We quantified changes in jawbone dimensions by measuring the distance from furcation (F) to intersecting points on incisors (FA′, FB′, and FC′) normalized to the fixed root length (FA, FB, and FC) (Fig. 1*E*). We observed significant jawbone expansion in *Sh3bp2* ^*KI/+*^ and *Sh3bp2* ^*KI/KI*^ but not in *Sh3bp2* ^*+/+*^ mice, even after pulp exposure for 42 days (Fig. 1*E*). These data suggest that CBM mutant SH3BP2 led to exacerbated inflammatory bone loss in apical periodontitis. *Sh3bp2* ^*KI/KI*^ and *Sh3bp2* ^*KI/+*^ mice, for the first time, replicate the human CBM expansile jawbone phenotype in a temporally and spatially defined manner. *Sh3bp2* ^*KI/+*^ mice required a longer infection time to develop jawbone expansion.

### Tlr2/4 signaling mediated the expansion of CBM‐like lesions

**Fig. 2:**
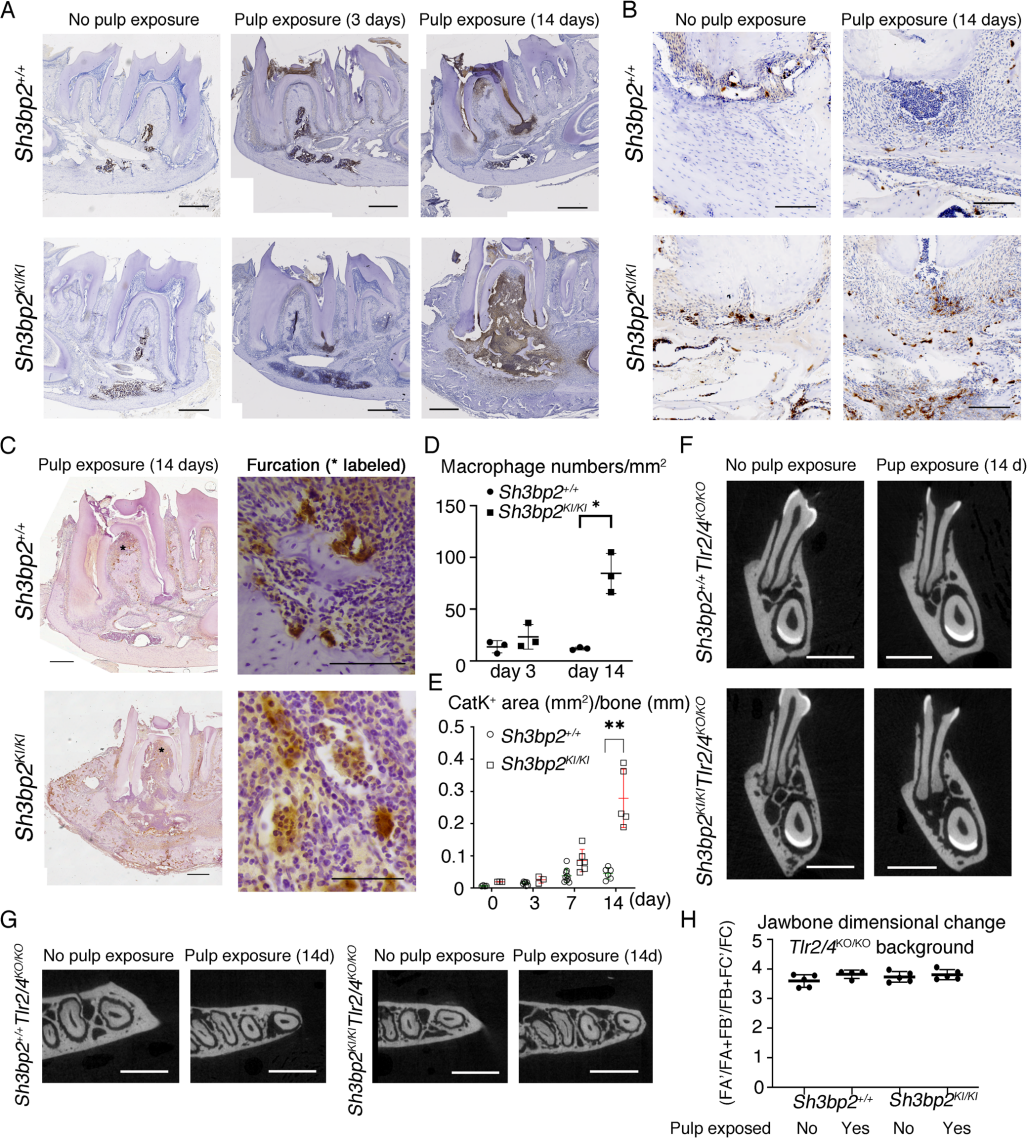
*Tlr2/4*‐mediated inflammatory bone resorption is exacerbated in *Sh3bp2* ^KI/KI^ mice. Immunohistochemistry of paraffin‐embedded *Sh3bp2* ^*KI/KI*^ mandibular sections show (*A*) rapid infiltration (3 days after pulp exposure, middle panel) and interspersed influx (14 days after pulp exposure, right panel) of neutrophils stained with NIMPR14 antibody. Neutrophils in *Sh3bp2* ^*+/+*^ mice were confined to the periapex of the roots. Scale bar = 500 μm. (*B*) Increased macrophages stained by CD68 antibody. Scale bar = 200 μm. (*C*) Increased bone resorption and multinucleated osteoclasts stained by CatK antibody. Scale bar = 500 μm (left) and 50 μm (right panel). (*D*) Quantification of macrophage numbers normalized to bone surface (mm^2^) and (*E*) CatK‐positive area normalized to bone perimeter (mm). *n* = 3–9 per group. (*F*) Coronal planes and (*G*) axial planes of μCT images from *Sh3bp2* ^*+/+*^ *Tlr2/4* ^*KO/KO*^ and *Sh3bp2* ^*KI/KI*^ *Tlr2/4* ^*KO/KO*^ mandibles with or without pulp exposure. (*H*) Quantitation of jawbone dimensions. No significant jawbone expansion 14 days after pulp exposure in *Sh3bp2* ^*KI/KI*^ mice when Tlr2/4 were knocked out although periapical lesions could still be observed. Data are presented as mean ± SD. Each dot in graphs represents a single biological sample (*n* = 4–5 per group). Measurements were analyzed using two‐way ANOVA followed by Sidak correction. *p < 0.05, **p < 0.01 indicate statistical significance. CatK = cathepsin K.

To elucidate molecular mechanisms leading to CBM‐like lesions, we compared *Sh3bp2* ^*+/+*^ and *Sh3bp2* ^*KI/KI*^ mice with or without pulp exposure for 14 days. We first examined the presence of neutrophils, macrophages, and osteoclasts in the periapices of mandibular 1st molars by immunostaining with NIMPR14, CD68 and cathepsin K (CatK) antibodies, respectively. We observed rapid neutrophil infiltration in the apical portion of pulp and periapex of the 1st molar in *Sh3bp2* ^*KI/KI*^ mice 3 days after pulp exposure, whereas in *Sh3bp2* ^*+/+*^ mice infiltration was limited to the coronal pulp (Fig. 2*A*, Supplemental Fig. S2 *A*). At day 14 of pulp exposure, neutrophils were constrained to root canals and periapices in *Sh3bp2* ^*+/+*^ mice, whereas neutrophils were interspersed in mandibles of *Sh3bp2* ^*KI/KI*^ mice (Fig. 2*A*). We next determined macrophage and osteoclast phenotype by normalizing macrophage numbers to the periapical surface area of 1st molars and osteoclast surface areas to alveolar bone perimeter surrounding 1st molars, respectively. Macrophage numbers and osteoclast surface areas were increased in *Sh3bp2* ^*KI/KI*^ mice 14 days after pulp exposure (Fig. 2*B*–*E*). Loss of furcal bone was consistent with increased osteoclasts in *Sh3bp2* ^*KI/KI*^ mice (Fig. 2*C*). Osteoclasts were distributed throughout the entire right treated mandible of *Sh3bp2* ^*KI/KI*^ mice, whereas they were confined to the area surrounding the 1st molar in *Sh3bp2* ^*+/+*^ mice (Fig. 2*C*, Supplemental Fig. S2 *B*).

Hosts recognize PAMPs and DAMPs primarily via Tlr2/4, which subsequently leads to inflammatory reactions.^(^ ^20^ ^)^ Therefore, we examined whether deletion of Tlr2/4 signaling in *Sh3bp2* mutant mice ameliorates PAMPs‐induced jawbone expansion. We crossed *Sh3bp2* ^*KI/+*^ mice with *Tlr2* ^*−/–*^ *Tlr4* ^*lps–del/lps–del*^ double knockout (*Tlr2/4* ^*KO/KO*^) mice to obtain *Sh3bp2* ^*+/+*^ *Tlr2/4* ^*KO/KO*^ and *Sh3bp2* ^*KI/KI*^ *Tlr2/4* ^*KO/KO*^ mice. Pulp exposure for 14 days did not lead to significant jawbone expansion in *Sh3bp2* ^*KI/KI*^ *Tlr2/4* ^*KO/KO*^ mice shown by μCT and Faxitron images (Fig. 2*F*–*H*, Supplemental Fig. S3 *A*). Although both *Sh3bp2* ^*+/+*^ *Tlr2/4* ^*KO/KO*^ and *Sh3bp2* ^*KI/KI*^ *Tlr2/4* ^*KO/KO*^ mice still developed periapical lesions, the inflammatory bone loss was reduced compared to mice with normal *Tlr2/4*. Pulp exposure for 14 days led to a tendency of increased numbers of macrophages and osteoclasts in *Sh3bp2* ^*KI/KI*^ *Tlr2/4* ^*KO/KO*^ mice but this increase was only statistically significant in *Sh3bp2* ^*KI/KI*^ mice without deletion of Tlr2/4, as evaluated by CD68 and cathepsin K immunohistochemistry (Supplemental Fig. S3 *B*,*C*). Taken together, our data suggest that *Sh3bp2* ^*KI/KI*^ mice develop CBM‐like jawbone expansion and exacerbated inflammatory bone resorption, which are at least in part mediated via Tlr2/4 signaling.

### Local and systemic inflammatory cytokines increased in *Sh3bp2* ^*KI*^ ^*/KI*^ mice after pulp exposure

**Fig. 3:**
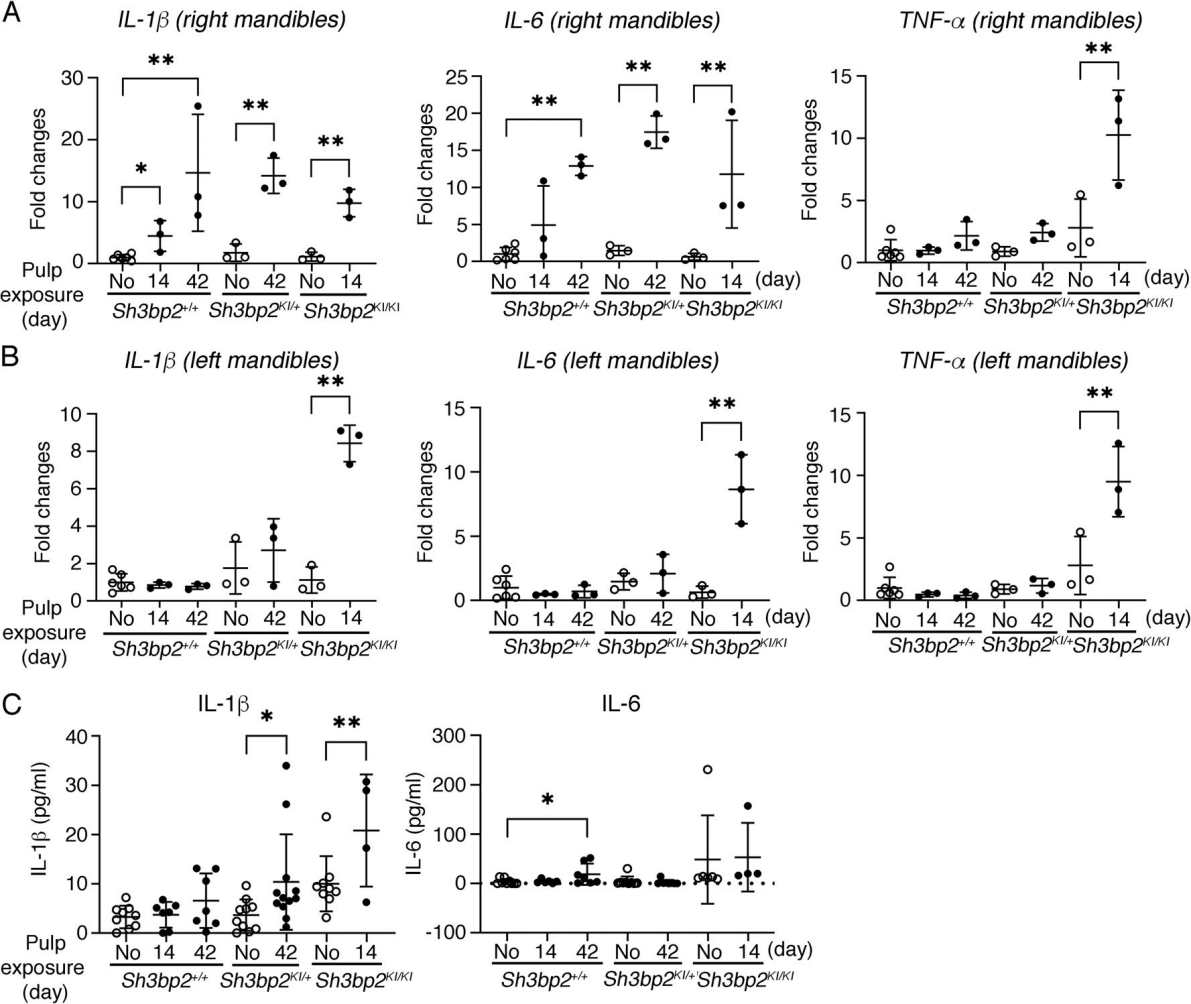
Increased local and systemic levels of inflammatory cytokines in *Sh3bp2* ^KI/KI^ mice after pulp exposure. qPCR showing gene expression of *IL‐1β*, *IL‐6*, and *TNF‐α* with or without pulp exposure (*A*) in treated right mandibles; (*B*) in left undrilled mandibles of *Sh3bp2* ^*+/+*^, *Sh3bp2* ^*KI/+*^, and *Sh3bp2* ^*KI/KI*^ experimental mice (*n* = 3–6 per group). (*C*) IL‐1β and IL‐6 plasma levels with or without pulp exposure in *Sh3bp2* ^*+/+*^, *Sh3bp2* ^*KI/+*^, and *Sh3bp2* ^*KI/KI*^ mice (*n* = 4–12 per group). No: naïve mice (no pulp exposure). Each dot in graphs represents a single biological sample. Data presented as mean ± SD. *p < 0.05, ** < 0.01 indicate significant difference by two‐way ANOVA followed by Sidak correction.

*IL‐1β*, *IL‐6*, and *TNF‐α* are the most common inflammatory cytokines detected in periapical lesions.^(^ ^13^ ^)^ We first examined their expression in alveolar bones surrounding right 1st mandibular molars after pulp exposure (right mandibles) by qPCR. Pulp exposure resulted in significantly increased levels of *IL‐1β* and *IL‐6* in right mandibles from *Sh3bp2* ^*+/+*^, *Sh3bp2* ^*KI/+*^, and *Sh3bp2* ^*KI/KI*^ mice compared to naïve mice (no pulp exposure) (Fig. 3*A*). *TNF‐α* was also increased in right mandibles of *Sh3bp2* ^*KI/KI*^ mice after pulp exposure but not in *Sh3bp2* ^*+/+*^ and *Sh3bp2* ^*KI/+*^ mice (Fig. 3*A*).

We next compared the left untreated mandibles of the experimental groups (pulp exposed on the right mandibles) to left mandibles from naïve mice, whose molars were not drilled on either side. Unexpectedly, *IL‐1β*, *IL‐6*, and *TNF‐α* were significantly increased on the left side of mandibles in *Sh3bp2* ^*KI/KI*^ mice despite pulp exposure only in right 1st molars (Fig. 3*B*). Cytokine expression in *Sh3bp2* ^*KI/+*^ mice showed an increasing but not significant trend in the left mandible (Fig. 3*B*). Moreover, plasma IL‐1β levels in *Sh3bp2* ^*KI/+*^ and *Sh3bp2* ^*KI/KI*^ mice were significantly increased after pulp exposure but not in *Sh3bp2* ^*+/+*^ mice (Fig. 3*C*). We were unable to detect significant changes in plasma IL‐6 level by ELISA assays in *Sh3bp2* ^*KI/+*^ and *Sh3bp2* ^*KI/KI*^ mice (Fig. 3*C*). These data suggest that PAMPs‐induced periapical lesions produced systemic inflammatory events in *Sh3bp2* ^*KI/KI*^ mice but were limited to the local environment in *Sh3bp2* ^*+/+*^ mice.

### Depletion of neutrophils ameliorates CBM‐like jawbone expansion in *Sh3bp2* ^*KI*^ ^*/KI*^ mice

**Fig. 4:**
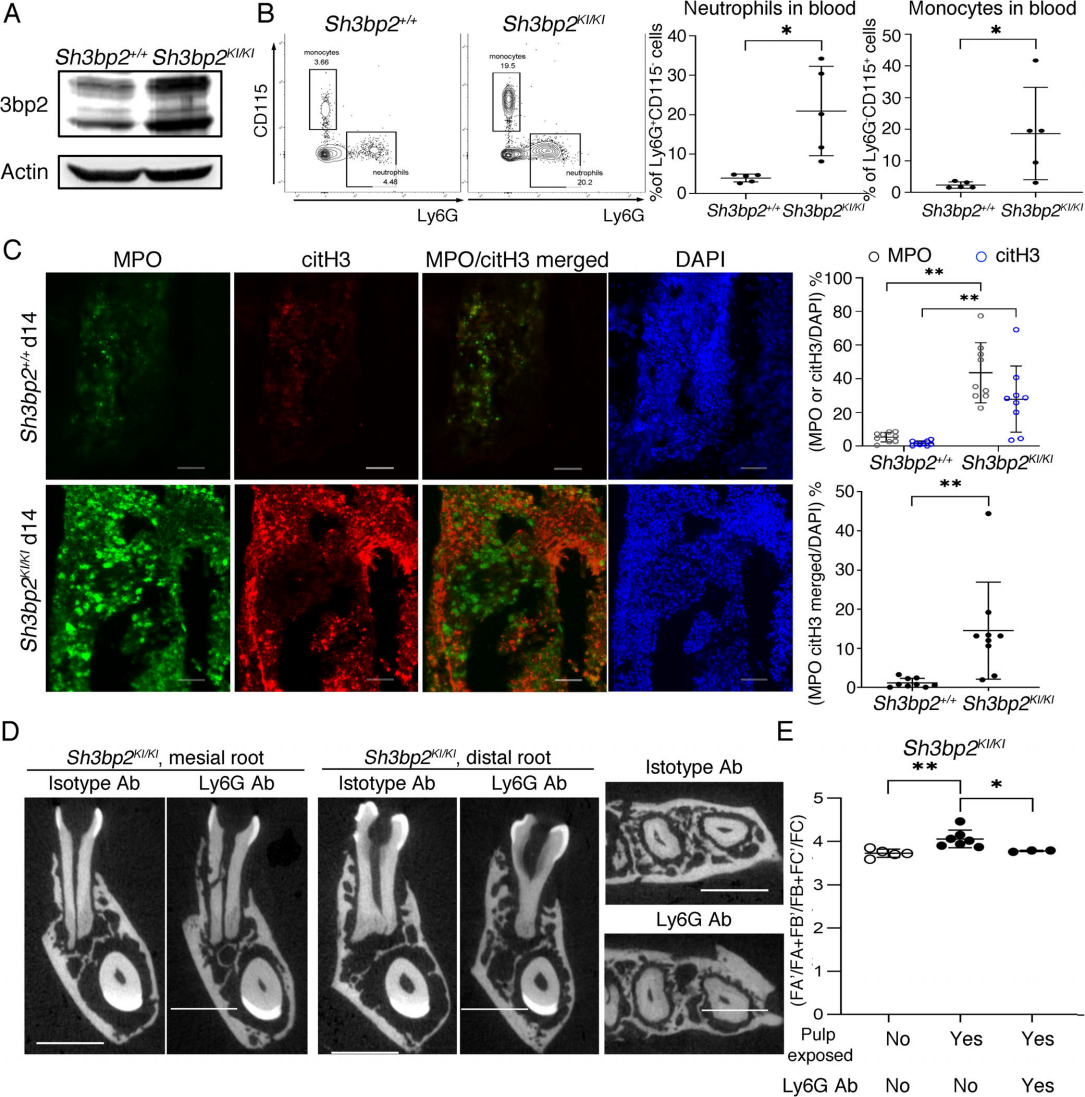
CBM mutation in *Sh3bp2* enhances neutrophil‐mediated events that contribute to PAMPs‐induced jawbone expansion. (*A*) Increased SH3BP2 expression in sorted Ly6G‐positive cells from *Sh3bp2* ^*KI/KI*^ bone marrow shown by immunoblotting. (*B*) Increased percentages of blood neutrophils (Ly6G^+^CD115^−^) and monocytes (Ly6G^−^CD115^+^) in *Sh3bp2* ^KI/KI^ mice shown by flow cytometry (*n* = 5 per group). (*C*) Detection of MPO (green) and citrullinated H3 (pink) in *Sh3bp2* ^*+/+*^ and *Sh3bp2* ^*KI/KI*^ right mandibles 14 days after pulp exposure. Scale bar = 200 μm. Histograms show quantification of MPO, citH3, and colocalization of MPO and citH3 signals normalized to DAPI (blue) from nine periapical and furcation areas in three *Sh3bp2* ^*+/+*^ and *Sh3bp2* ^*KI/KI*^ mice. (*D*) Coronal and axial planes of μCT images of *Sh3bp2* ^*KI/KI*^ mice treated with Ly6G antibody or isotype control prior to and after pulp exposure. (*E*) Quantification of mandible expansion shows significant amelioration after Ly6G antibody treatment in *Sh3bp2* ^*KI/KI*^ mice compared to no antibody or isotype control (arrows) antibody. Scale bar = 1 mm. Each dot in graphs represents a single biological sample (*n* = 3–7 per group). Data are presented as mean ± SD. *p < 0.05, **p < 0.01 indicate significant difference by Student's *t* test (*B*), (*C*) and one‐way ANOVA (*E*).

Rapid and intensive infiltration of neutrophils following pulp exposure in *Sh3bp2* ^*KI/KI*^ mice prompted us to examine the role of neutrophils in the pathogenesis of CBM. To examine whether SH3BP2 is expressed in neutrophil‐enriched cell populations, cell lysate of sorted Ly6G‐positive neutrophils in bone marrow was subjected to SH3BP2 immunoblotting. SH3BP2 levels were significantly increased in Ly6G‐positive cells from *Sh3bp2* ^*KI/KI*^ mice compared to *Sh3bp2* ^*+/+*^ mice (Fig. 4*A*). These data are consistent with increased SH3BP2 levels in bone marrow–derived macrophages, lymph node, thymus, and spleen of *Sh3bp2* ^*KI/KI*^ mice.^(^ ^8^, ^9^, ^21^ ^)^ We next assessed neutrophil counts in blood from untreated *Sh3bp2* ^*+/+*^ and *Sh3bp2* ^*KI/KI*^ mice by flow cytometry. The percentages of neutrophils (Ly6G^+^CD115^−^) and monocytes (Ly6G^−^CD115^+^) in blood were significantly increased in *Sh3bp2* ^*KI/KI*^ compared to *Sh3bp2* ^*+/+*^ mice (Fig. 4*B*). NETs can be detected by co‐staining with antibodies for citH3 and neutrophil‐derived proteins MPO or neutrophil elastase.^(^ ^22^ ^)^ Levels of MPO, citH3, and NETs formation (co‐localization of MPO and citH3) in jawbones were significantly increased in *Sh3bp2* ^*KI/KI*^ mice compared to *Sh3bp2* ^*+/+*^ mice with pulp exposure (Fig. 4*C*). MPO expression appeared earlier and more widely than citH3‐positive staining and the intensity of MPO and citH3 staining increased from day 3 to day 14 after pulp exposure (Supplemental Fig. S4 *A*,*B*). Furthermore, plasma level of MPO was also significantly increased in *Sh3bp2* ^*KI/KI*^ mice after pulp exposure (Supplemental Fig. S4 *C*).

To examine whether neutrophil depletion ameliorates the CBM‐like jawbone expansion induced by PAMPs, we adapted previously published protocols by injecting *Sh3bp2* ^*KI/KI*^ mice intraperitoneally with Ly6G‐specific antibody (1A8; Bio X Cell, Lebanon, NH, USA) at 350 μg 24 hours before pulp exposure followed by 200 μg every 2 days for 14 days.^(^ ^23^, ^24^, ^25^, ^26^, ^27^ ^)^ Sham control *Sh3bp2* ^*KI/KI*^ mice received pulp exposure and were injected with isotype control antibody (RatIgG2a; BioXCell) following the same regimen as the treatment group. Mice that received Ly6G‐specific antibody showed massive reduction of neutrophils in the periapical lesions when compared to mice injected with isotype IgG control antibody (Supplemental Fig. S5 *A*). Faxitron and μCT images showed that Ly6G treatment of *Sh3bp2* ^*KI/KI*^ mice attenuated bone loss and jawbone expansion induced by pulp exposure (Fig. 4*D* and Supplemental Fig. S5 *B*). Changes in jawbone dimensions of *Sh3bp2* ^*KI/KI*^ mice that received pulp exposure and Ly6G antibody treatment were not significantly different from mandibles of naïve *Sh3bp2* ^*KI/KI*^ mice without pulp exposure (Fig. 4*E*). These results suggest that SH3BP2‐mutant neutrophils play an important role in jawbone expansion of *Sh3bp2* ^*KI/KI*^ mice and that neutrophil‐mediated events may be promising therapeutic targets to reduce progression of CBM.

## Discussion

There are still several knowledge gaps and experimental challenges in CBM research, which are in part due to species differences between humans and mice. SH3BP2 KI mice do not spontaneously develop jawbone expansion, which is the characteristic feature of CBM patients. In human, CBM occurs as an autosomal dominant trait whereas *Sh3bp2* ^*KI/+*^ mice are phenotypically closer to *Sh3bp2* ^+/+^ mice.^(^ ^6^ ^)^ Another challenge is the lack of human samples, especially in developing stages or before the onset of CBM lesions are noticed. All published studies examine human mature CBM lesions excised during surgery. We have improved the existing CBM mouse model to be more clinically relevant. After pulp exposure, both *Sh3bp2* ^*KI/KI*^ and *Sh3bp2* ^*KI/+*^ mice develop jawbone expansion in a defined location and predictable time course. This research tool allows us to study cellular and molecular abnormalities during the activation stage of CBM‐like lesions, investigate the response to inflammatory stimuli, and validate potential therapeutic strategies for CBM.

Jaw and oral tissues are exposed to bacterial flora and occlusal load, which are considered PAMPs and DAMPs. It has been proposed that PAMPs and DAMPs significantly affect the Tlr2/4‐mediated immune system.^(^ ^20^, ^28^, ^29^ ^)^ In the absence of Tlr2/4, autoinflammatory lesions of *Sh3bp2* ^*KI/KI*^ mice are rescued.^(^ ^10^ ^)^ Our study further suggests that PAMPs recognition by Tlr2/4 in the oral cavity is largely responsible for the development of the CBM‐like jawbone expansion. *Sh3bp2* ^*KI/KI*^ *Tlr2/4* ^*KO/KO*^ mice not only did not show expansile jawbones but also did not lose weight after pulp exposure (data not shown), unlike the continuous weight loss seen in *Sh3bp2* ^KI/KI^ mice. Body weight loss after dental drilling has been associated with toothache.^(^ ^30^ ^)^ We speculate that *Sh3bp2* ^*KI/KI*^ mice develop painful apical periodontitis after pulp exposure and knocking out TLR2/4 in *Sh3bp2* ^*KI/KI*^ mice may lessen their pain levels. It is possible that CBM‐like lesions and mandible expansion in *Sh3bp2* ^*KI/KI*^ *Tlr2/4* ^*KO/KO*^ mice may be delayed beyond our observation period and involve other molecular signaling pathways such as the nucleotide‐binding oligomerization domain 1 and 2 (NOD1/2) pathway.

Although CBM lesions can be unilateral, most CBM patients show bilateral swelling of the mandible and/or the maxilla, even more extensively in zygomatic arches, condyles, or ribs.^(^ ^2^, ^31^, ^32^ ^)^ A recent case report showed evidence of systemic inflammation in a CBM patient.^(^ ^33^ ^)^ This is in accordance with our finding that inflammatory cytokines are locally and systemically upregulated in *Sh3bp2* ^*KI/KI*^ mice during jawbone expansion. It is plausible that *Sh3bp2* ^*KI/KI*^ mice may develop bilateral jawbone expansion with longer infection time (>14 days) due to the systemic inflammation. These finding have clinical implications for CBM as well as for endodontic infection. Systemic increase of inflammatory cytokines may contribute to the bilateral jawbone expansion in CBM patients. The data from *Sh3bp2* ^*+/+*^ mice are consistent with the current dogma that endodontic infection is limited to local environment. However, findings from *Sh3bp2* ^*KI/KI*^ mice suggest that precautionary measures such as the use of prophylactic antibiotics might be appropriate before endodontic treatment of patients with CBM or other autoimmune disorders to control and prevent systemic infection.

Neutrophils, the most abundant white blood cell type, are the first line of immune defense against invading pathogens via phagocytosis, degranulation, production of reactive oxygen species (ROS), and the release of NETs. Neutrophils have been shown to play important roles in the pathogenesis of various systemic autoimmune diseases, such as adult‐onset Still's disease, rheumatoid arthritis, and systemic lupus erythematosus,^(^ ^34^, ^35^, ^36^ ^)^ but not CBM. We found that mouse CBM‐like lesions have an amplified inflammatory reaction with fast infiltration of numerous neutrophils, prior to increased numbers of macrophages and osteoclasts. Depletion of neutrophils ameliorated jawbone expansion in CBM mice further supports the important role of neutrophils in the pathogenesis of CBM. We propose that upon the stimulation with PAMPs or DAMPs, CBM mutant neutrophils release NETs and cytokines, which prompt macrophage recruitment to engulf NETs and activate other immune cells leading to a cytokine burst. The infiltration of neutrophils at CBM‐like lesions may be enhanced by the abundance of neutrophils and monocytes in blood of *Sh3bp2* ^*KI/KI*^ mice at baseline. Future studies will investigate how SH3BP2 accumulation affects development, maturation, function, and neutrophil apoptosis by PAMPs or DAMPs.

To date, there are no biomarkers with sufficient sensitivity and specificity to diagnose, monitor disease progression, or predict disease severity and prognosis for CBM. Human studies provided some reports that serum levels of alkaline phosphatase or phosphate increased during the activation stage of CBM.^(^ ^37^, ^38^, ^39^ ^)^ However, these increases are also seen in many other bone diseases. Based on our results, we propose that an increased level of several cytokines (*IL‐1β*, *IL‐6*, and *TNF‐α*), NETs markers (MPO levels), and markers of increased neutrophil numbers or function could be potential biomarkers for the activation stage of CBM‐like jawbone expansion. Our method also has the potential to identify biomarkers for CBM jawbone expansion at different stages, including activation, amplification, and regression, with more elaborate analysis.

In summary, we demonstrated that *Sh3bp2* ^*KI/+*^ and *Sh3bp2* ^*KI/KI*^ mice replicate human CBM jawbone expansion when challenged by eliciting PAMPs. In addition to known bony inflammatory events (increased numbers of macrophages, osteoclasts, and bone resorption) in CBM lesions, our study reveals a previously unrecognized role of neutrophils that may play a vital role in promoting CBM lesions. The significantly increased cytokines and MPO level in *Sh3bp2* ^*KI/KI*^ mice exhibiting jawbone expansion suggests potential biomarkers for CBM.

## Conflict of Interest

All authors declare that they have no conflicts of interest.

### Peer Review

The peer review history for this article is available at https://publons.com/publon/10.1002/jbm4.10562.

## Supporting information


**Appendix S1**. Supporting Information
