# Supplementary material for: Tlr2/4‐Mediated Hyperinflammation Promotes Cherubism‐Like Jawbone Expansion in Sh3bp2 (P416R) Knockin Mice
Source: JBMR Plus. 2021 Oct 30;6(1):e10562. doi: 10.1002/jbm4.10562 (PMC8771001; doi:10.1002/jbm4.10562)
Supplement: Supplementary file 1 — Appendix S1. Supporting Information [file JBM4-6-e10562-s001.pdf]

Tlr2/4-mediated Hyperinflammation Promotes Cherubism-like Jawbone Expansion in Sh3bp2 (P416R) Knockin Mice

Yasuyuki Fujii[1], Nelson Monteiro[1], Shyam Kishor Sah[1], Homan Javaheri[1], Yasuyoshi Ueki[2,3], Zhichao Fan[4], Ernst J Reichenberger[5], and I-Ping Chen[1]*

[1]Department of Oral Health and Diagnostic Sciences, School of Dental Medicine, University of Connecticut Health, Farmington, CT, United States.
[2]Department of Biomedical Sciences and Comprehensive Care, Indiana University School of Dentistry, Indianapolis, IN, United States.
[3]Indiana Center for Musculoskeletal Health, Indiana University, School of Medicine, Indianapolis, IN, United States.
[4]Department of Immunology, School of Medicine, University of Connecticut Health, Farmington, CT, United States.
[5]Center for Regenerative Medicine and Skeletal Development, Department of Reconstructive Sciences, University of Connecticut Health, Farmington, CT, United States.

***Corresponding author:**
I-Ping Chen
Department of Oral Health and Diagnostic Sciences
University of Connecticut Health
263 Farmington Avenue, Farmington, CT 06030, United States
Tel.: +1-860-679-1030
E-mail address: ipchen@uchc.edu

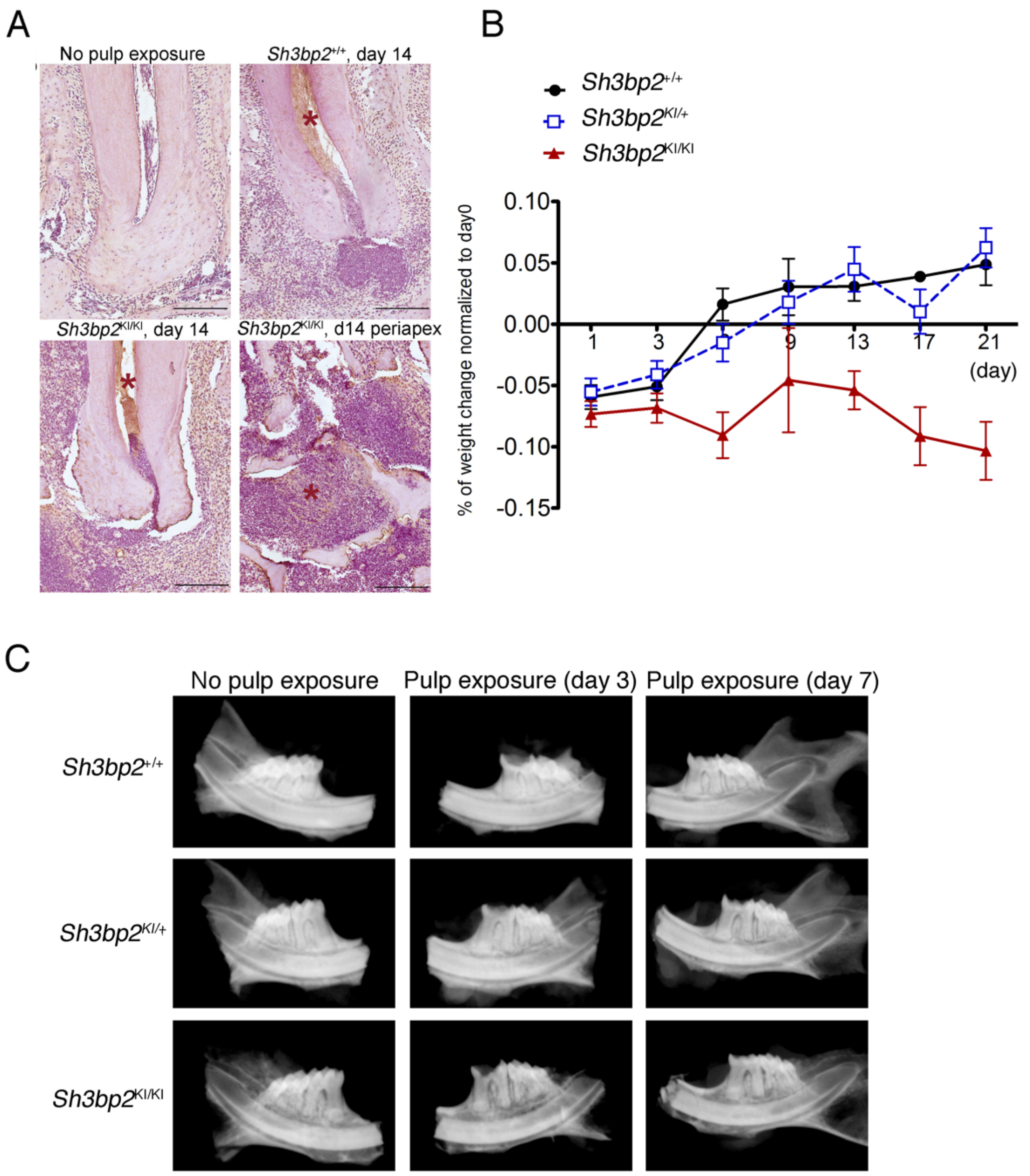

Supplemental Figure 1: Introducing PAMPs into jawbone by pulp exposure of first mandibular molar. **(A)** The presence of lipopolysaccharides (LPS) in root canals and periapices 14 days after pulp exposure. Scale bar = 200 µm. * indicates positive LPS core brown staining. **(B)** Body weight changes in *Sh3bp2*$^{+/+}$ (black line), *Sh3bp2*$^{KI/+}$ (blue line), and *Sh3bp2*$^{KI/KI}$ (red line) mice after pulp exposure; n = 4-13 per time point per group. **(C)** Representative Faxitron images of mandibles with and without pulp exposure for 3 and 7 days from *Sh3bp2*$^{+/+}$, *Sh3bp2*$^{KI/+}$, and *Sh3bp2*$^{KI/KI}$ mice.

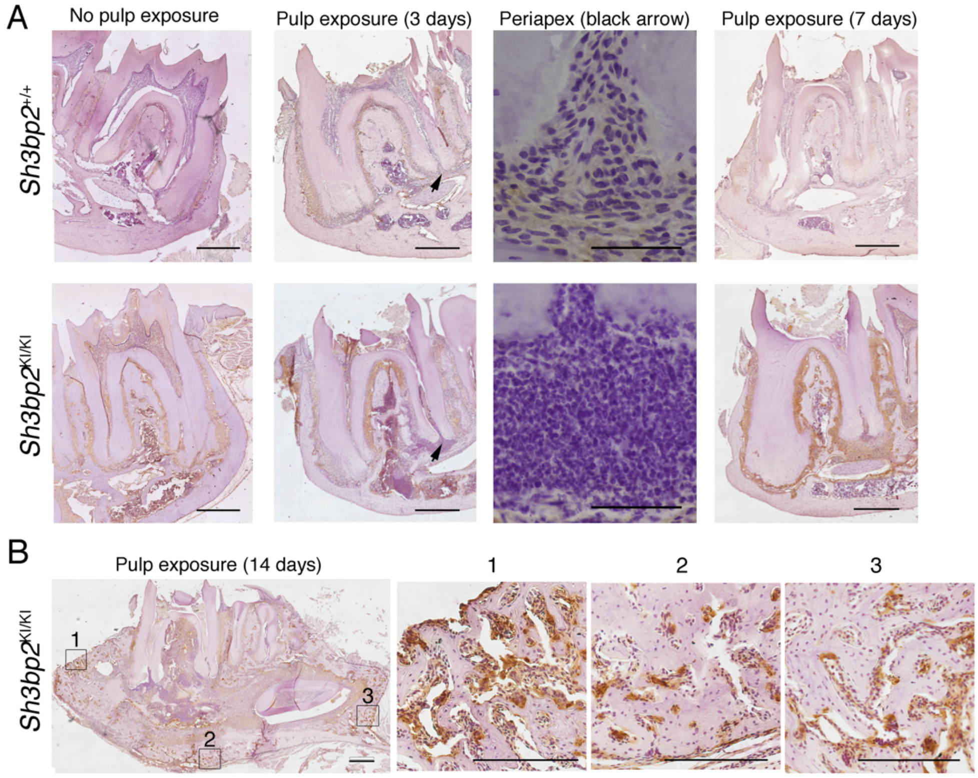

Supplemental Figure 2: Inflammatory bone resorption after pulp exposure in *Sh3bp2*$^{+/+}$ and *Sh3bp2*$^{KI/KI}$ mice. **(A)** Inflammatory cells rapidly appear in the periapex of *Sh3bp2*$^{KI/KI}$ mice 3 and 7 days after pulp exposure. Enlarged H&E staining images of periapices at day 3 (indicated by black arrow heads) show differences in cellular morphology and cell density between *Sh3bp2*$^{+/+}$ and *Sh3bp2*$^{KI/KI}$ mice. Periapex of *Sh3bp2*$^{+/+}$ mice at day 3 and mandibles from *Sh3bp2*$^{+/+}$ and *Sh3bp2*$^{KI/KI}$ mice without pulp exposure show no obvious signs of inflammation. Scale bar = 500 µm (1st, 2nd and 4th panels from the left); Scale bar = 50 µm (3rd panel). **(B)** Diffuse bone resorption and interspersed osteoclasts (brown-stained multinucleated cells) 14 days after pulp exposure in *Sh3bp2*$^{KI/KI}$ mice shown by CatK immunohistochemistry. Enlarged images 1, 2, and 3 of areas indicated by square boxes in left panel. Note: CatK positive cells were observed in front margin (box 1), inferior border (box 2) and the posterior end (box 3) of mandibles. Scale bar = 500 µm (left panel); Scale bar = 250 µm (2nd, 3rd, and 4th panels from the left).

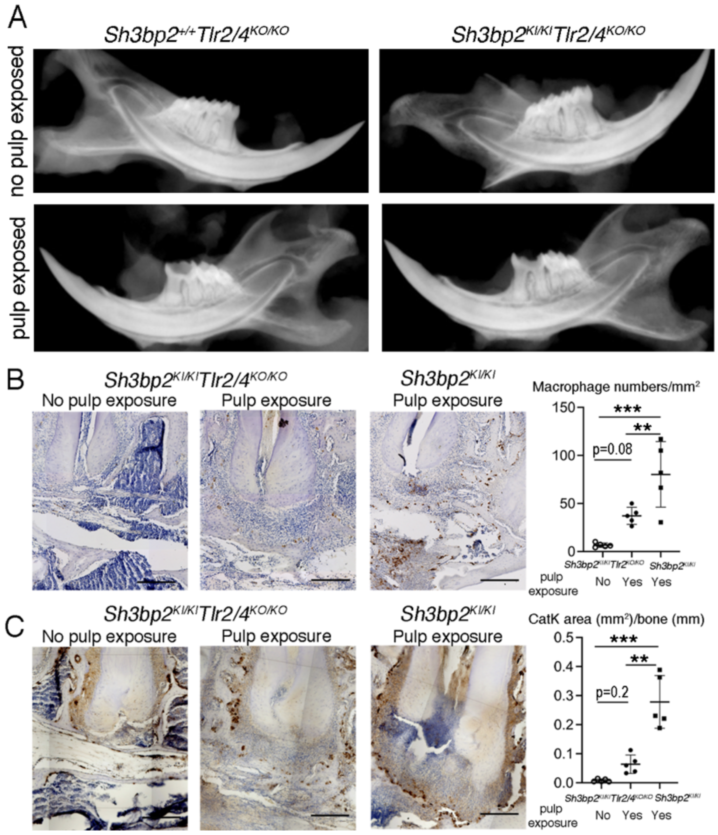

Supplemental Figure 3: Mitigation of inflammatory responses in *Sh3bp2*$^{KI/KI}$ mice with *Tlr2/4*$^{KO/KO}$ background after pulp exposure. (**A**) Representative Faxitron images of mandibles with and without pulp exposure for 14 days of *Sh3bp2*$^{+/+}$*Tlr2/4*$^{KO/KO}$ and *Sh3bp2*$^{KI/KI}$ *Tlr2/4*$^{KO/KO}$ mice; (**B**) Immunohistochemistry of CD68 and (**C**) Cathepsin K of paraffin-embedded mandibular sections from *Sh3bp2*$^{KI/KI}$*Tlr2/4*$^{KO/KO}$ mice with and without pulp exposure as well as from *Sh3bp2*$^{KI/KI}$ mice (n=3-5). Scale bar = 200 µm. Quantification of macrophage numbers normalized to bone surface (mm^2) and CatK-positive area normalized to bone perimeter (mm). Measurements were analyzed using one-way ANOVA followed by Tukey's multiple comparison test. **$p<0.01$, ***$p<0.001$ indicate statistical significance.

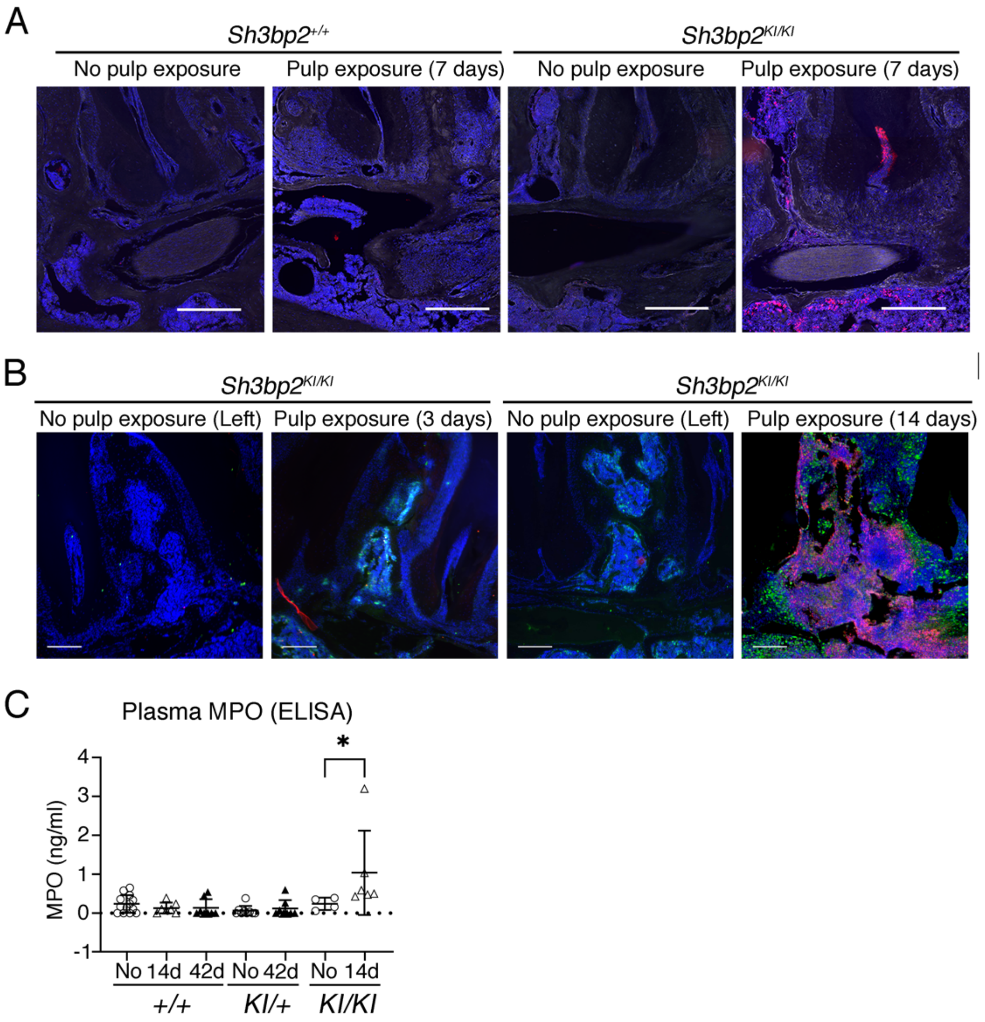

Supplemental Figure 4: (**A**) Detection of citrullinated H3 (pink) in *Sh3bp2*$^{KI/KI}$ mandibles 7 days after pulp exposure. DAPI nuclear staining: blue. Scale bar = 500 µm. (**B**) NETs formation increased over time in *Sh3bp2*$^{KI/KI}$ mice after pulp exposure shown by immunostaining with MPO (green) and citrullinated H3 (pink) antibodies. DAPI nuclear staining: blue. Scale bar = 200 µm. (**C**) Plasma level of MPO is increased only in *Sh3bp2*$^{KI/KI}$ mice 14 days after pulp exposure. No: naïve mice with no pulp exposure. +/+: *Sh3bp2*$^{+/+}$, *KI*/+: *Sh3bp2*$^{KI/+}$, *KI/KI*: *Sh3bp2*$^{KI/KI}$ mice. Data are presented as mean ± S.D. Each dot in graphs represents a single biological sample (n=4-13 per group). Measurements were analyzed using two-way ANOVA followed by Sidak correction. *$p<0.05$ indicates statistical significance.

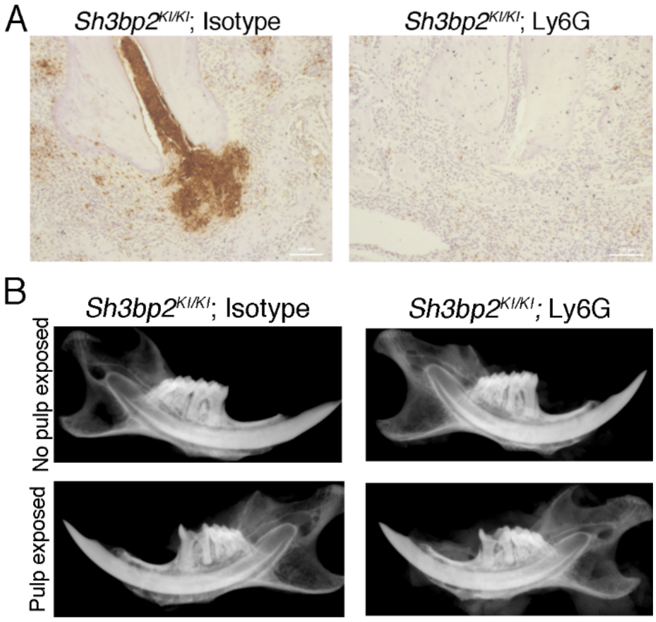

Supplemental Figure 5: (**A**) Ly6G antibody, but not isotype control antibody treatment, depleted neutrophils in the periapical area after pulp exposure for 14 days in *Sh3bp2*$^{KI/KI}$ mice shown by NIMPR14 immunohistochemistry. Scale bar = 100 μm; (**B**) Representative Faxitron images of mandibles with and without pulp exposure for 14 days from *Sh3bp2*$^{+/+}$ mice injected with isotype control or Ly6G antibodies.

Supplemental table 1. Sequence information of primers used for quantitative qPCR

Gene	Primer sequences
Mouse IL-1β	Forward: 5'-CAACCAACAAGTGATATTCTCCATG-3'
	Reverse: 5'-GATCCACACTCTCCAGCTGCA-3'
Mouse IL-6	Forward: 5'-CAACGATGATGCACTTGCAG-3'
	Reverse: 5'-GAAATTGGGGTAGGAAGGAC-3'
Mouse TNF-α	Forward: 5'-CGCTCTTCTGTCTACTGAAC-3'
	Reverse: 5'-TGTCCCTTGAAGAGAACCTG-3'
Mouse 18S	Forward: 5'-TTGACGGAAGGGCACCACCAG-3'
	Reverse: 5'-GCACCACCACCCACGGAATCG-3'
